# Clopidogrel, an ADP-P2Y12 Receptor Antagonist, Can Prevent Severe Postoperative Pain: A Retrospective Chart Review

**DOI:** 10.3390/life10060092

**Published:** 2020-06-22

**Authors:** Rikuhei Tsuchida, Masahiko Sumitani, Hiroaki Abe, Masae Ando, Kosuke Saita, Kohshi Hattori, Kazuhito Mietani, Reo Inoue, Kanji Uchida

**Affiliations:** 1 Department of Anesthesiology and Pain Relief Center, The University of Tokyo Hospital, Tokyo 113-0033, Japan; tsuchidar-ane@h.u-tokyo.ac.jp (R.T.); andom-ane@h.u-tokyo.ac.jp (M.A.); saitak-ane@h.u-tokyo.ac.jp (K.S.); khatsutori-tky@umin.org (K.H.); mietanik-ane@h.u-tokyo.ac.jp (K.M.); inouer-ane@h.u-tokyo.ac.jp (R.I.); uchidak-ane@h.u-tokyo.ac.jp (K.U.); 2 Department of Pain and Palliative Medicine, The University of Tokyo Hospital, Tokyo 113-0033, Japan; abeh-pla@h.u-tokyo.ac.jp

**Keywords:** ATP receptor, P2Y12 receptor, clopidogrel, postoperative pain, central sensitisation

## Abstract

The purinergic P2Y12 receptor regulates microglial activation, resulting in persistence and aggravation of pain in neuropathic and nociceptive pain models. We conducted a retrospective chart review to explore the analgesic potency of the P2Y12 receptor-specific antagonist, clopidogrel, for clinical management of postoperative pain in patients who underwent abdominal surgery. Twenty-seven patients with cardiovascular comorbidities, who underwent laparoscopic abdominal surgery and had ceased aspirin (ASP, *n* = 17) or clopidogrel (CLP, *n* = 10) for 14 days pre-operatively, were enrolled retrospectively. In both groups, the number of opioids and non-steroidal anti-inflammatory drugs (NSAIDs) consumed for managing postoperative pain was compared using the chi-square test and Mann–Whitney test. Our results showed that from postoperative day (POD) 0 to POD 3, the average numerical rating reflecting the postoperative pain was comparable between the two groups (CLP: 4.0 ± 1.4 vs. ASP: 3.7 ± 0.8, P-value = 0.56). However, at POD 7, opioid consumption in the CLP-treated group (fentanyl-equivalent dose: 0.49 ± 0.56 mg) was significantly lower than that in the ASP-treated group (1.48 ± 1.35 mg, P-value = 0.037). After reaching a stable state by repeated systemic administration, clopidogrel sustained the analgesic efficacy for a certain period. In conclusion, microglial P2Y12 receptors may mediate signal transduction of postoperative nociceptive pain and enhance clinical opioid analgesia.

## 1. Introduction

Pain is one of the most frequent symptoms observed in postoperative patients. Postoperative pain not only causes distress but also interferes with cough, breathing depth, and expectoration during the acute postoperative period and can be followed by atelectasis, pneumonia, and hypoxia [1]. Furthermore, postoperative pain can trigger the development of delirium and an associated increased mortality and morbidity [2]. Although a multimodal analgesic strategy is recommended to control postoperative pain, opioids still hold a prominent position. A valid concern is that along with the favourable positive effects of opioids (i.e., analgesia), the diverse negative side effects (e.g., nausea and vomiting, respiratory depression, and opioid-induced delirium) may also be triggered. Recently, it has been reported that opioids prescribed during acute postoperative periods could trigger misuse and dependence [3]. Therefore, as alternatives to opioids, novel analgesic agents are required to control postoperative pain.

Regarding the mechanisms of severe postoperative pain, central sensitisation is a possible target candidate for exploring novel agents. An emerging concept of central sensitisation states that immune cells (e.g., microglia) and neurons form an integrated network, in which activation of an immune response modulates the excitability of nociceptive pathways [4]. Microglia are activated when physiological homeostasis is threatened by several factors, including inflammation. This is followed by activated microglia potently modulating neuronal firing, resulting in hyperalgesia. Microglia express an array of purinergic receptors with important roles in activation, chemotaxis, process extension, and paracrine signalling in these cells [5]. Particularly, P2Y12 receptor expression is robust in the “resting” state but is substantially reduced after microglial activation. In the nervous system, the P2Y12 receptor is the primary site where nucleotides act to induce microglial activation during the initial response to threatened homeostasis [6]. Therefore, the P2Y12 receptor is considered a possible key regulator in preventing the aggravation of clinical pain. Previously, we have provided supporting evidence demonstrating that genetic polymorphisms of the P2Y12 receptor are closely linked to the aggravation of inflammatory pain, including postoperative pain and cancer pain [7]. Reportedly, genetic polymorphism of the P2Y12 receptor alters its functional property [8]. Apart from microglia, P2Y12 receptors are abundantly expressed on the platelet membrane and induce platelet aggregation when stimulated. Thienopyridine compounds such as clopidogrel are P2Y12 receptor-specific antagonists that can comprehensively inhibit platelet aggregation.

Based on this concept, we hypothesised that clopidogrel and its active metabolite may antagonise P2Y12 receptors not only on platelets but also on microglia at the spinal cord level, and thereby suppress pain through microglial inhibition. To the best of our knowledge, no report has documented the intimate relationship between clopidogrel and clinical pain. In this study, we conducted a retrospective chart review to explore the analgesic potency of clopidogrel for managing postoperative pain in patients who underwent abdominal surgery.

## 2. Results

### 2.1. Patient Background and Pain Assessment

**Figure 1:**
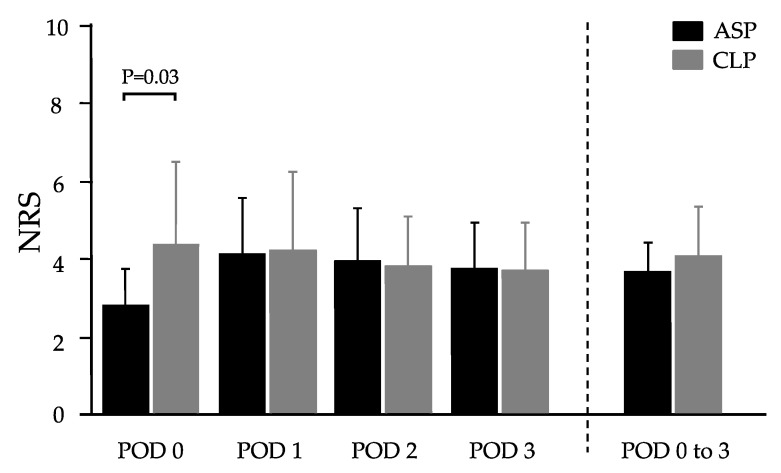
Postoperative pain intensity in patients who were administered aspirin or clopidogrel; numerical rating scale (NRS) for ASP and CLP. Values represent mean ± SD. A significant difference was observed only on postoperative day (POD) 0 (immediately after surgery) (P = 0.03). ASP, aspirin; CLP, clopidogrel.

We retrospectively reviewed the pharmacy and medical records of 27 patients with a history of cardiovascular diseases, who underwent laparoscopic abdominal surgery and were prescribed antiplatelet drugs. Patients taking clopidogrel within 14 days of cessation (CLP, N = 10); and those taking aspirin (ASP, N = 17) were compared. Table 1 presents patient backgrounds. No particular bias was observed in the background. All patients were administered general anaesthesia in combination with epidural anaesthesia. Postoperative pain was treated with a continuous epidural infusion of local anaesthetics and intravenous fentanyl. When pain deteriorated, either bolus intravenous fentanyl 10–30 µg/dose or non-steroidal anti-inflammatory drugs (NSAIDs) were administered at the discretion of the attending physician. Figure 1 shows the numerical rating scale (NRS; mean ± standard deviation) of postoperative pain at four timepoints (i.e., immediately after surgery, and postoperative days (PODs) 1 to 3) and the averaged NRS. Immediately after surgery, CLP patients complained of more severe pain (NRS = 4.4 ± 2.1) than ASP patients (NRS = 2.8 ± 1.0, P-value < 0.03). From POD 1 to POD 3, differences in postoperative pain intensity were not significant between the two groups. Overall, the average NRS of postoperative pain was comparable between the two groups (CLP: 4.0 ± 1.4 vs. ASP: 3.7 ± 0.8, P-value = 0.56).

### 2.2. Opioid Analgesic Required for Postoperative Pain

**Figure 2:**
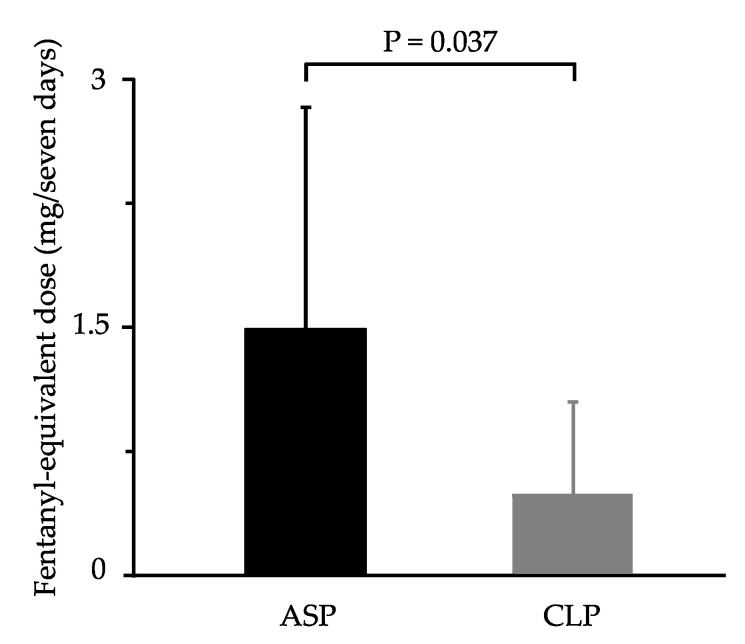
The total amount of opioid analgesics used within 7 days of surgery in two treatment groups. Values represent mean ± SD. A significant difference can be observed between ASP (1.48 ± 1.35 mg) and CLP (0.49 ± 0.56 mg, P-value = 0.037). ASP, aspirin; CLP, clopidogrel.

Figure 2 demonstrates the total amount of opioid analgesics administered until POD 7. For postoperative analgesia, the amount of opioid administered was significantly lower in CLP (0.49 ± 0.56 mg) than in ASP (1.48 ± 1.35 mg, P-value = 0.037). Additionally, the percentage of patients using NSAIDs during the postoperative period did not differ between groups. Thus, postoperative pain was comparable between the two groups, however, CLP patients required fewer opioids than ASP patients to control postoperative pain.

## 3. Discussion

The purpose of this study was to determine whether clopidogrel affected postoperative pain. Clopidogrel is a specific P2Y12 receptor antagonist, and possibly acts by antagonising microglial P2Y12 receptors, thus exerting an analgesic effect. When postoperative pain was similarly managed in both groups, during the 7 days post-surgery, the total amount of opioids administered was significantly higher in the aspirin group than in the clopidogrel group. This indicated that clopidogrel exerts potential analgesic effects in managing postoperative pain.

The P2Y12 receptor is expressed on the spinal cord microglia and is responsible for the initial stage of microglial activation, which is known to aggravate neuropathic and nociceptive pain [6,9]. In *P2Y12* gene knock-out mice, pain aggravation after microglial activation was not observed [10]. Previously, our findings have demonstrated that genetic polymorphisms of the P2Y12 receptor resulted in individual differences in cancer pain, as well as postoperative pain following abdominal surgery [7]. Thus, the P2Y12 receptor could be involved in regulating the severity of pain possibly through microglial activation. Reportedly, clopidogrel demonstrates potent analgesic efficacy. In a spinal nerve ligation neuropathic pain model, oral clopidogrel (10 mg/kg) increased the pain threshold and suppressed mechanical allodynia and thermal hyperalgesia in the injured limb of adult rats [10]. In the present study, we observed that patients administered with clopidogrel required fewer opioid analgesics for controlling postoperative pain than those treated with aspirin, indicating the pre-emptive-like analgesic effect of clopidogrel. Furthermore, in addition to the involvement of microglial activation in aggravated pain, it was also associated with opioid tolerability; this is attributed to microglia in the midbrain periaqueductal grey matter (PAG), and not microglia in the spinal cord. When activated, microglia produce and release an increased amount of pro-inflammatory cytokines, which can contribute to opioid tolerance [11]. Previously, some human studies have documented that pain intensity and opioid consumption may not be related [12,13]. Therefore, two possible underlying mechanisms can be postulated; (1) clopidogrel, the specific P2Y12 receptor antagonist, continues to antagonise the microglial P2Y12 receptor, thereby suppressing enhanced spinal transmission of postoperative pain by inhibiting microglial activation; and (2) clopidogrel suppresses microglial activation at the PAG and prevents opioid tolerance. Since clopidogrel has a potency of inhibiting platelet aggravation and bleeding is one of the major complications associated with clopidogrel intake, patients should stop clopidogrel therapy for a predetermined period before surgery to prevent postoperative haemorrhage. Therefore, clopidogrel is not used for postoperative pain management, and we failed to investigate the direct analgesic potency of clopidogrel for postoperative pain. We demonstrated its pre-emptive-like analgesic potency in this study although clopidogrel is not suitable as an adjuvant analgesic for postoperative pain. Notably, clopidogrel is an irreversible antagonist of the P2Y12 receptor; once it binds to the P2Y12 receptor, it remains bound during platelet survival. However, the pharmacodynamics–pharmacokinetics of clopidogrel on neural substrates, as well as the lifespan and turnover of microglia in the human spinal cord and midbrain, remain unclear. In the nervous system, most immune cells do not live longer than a few days or weeks [14,15]. In another study, microglial turnover was found to vary by the brain region in mice, for example, 41 months in the cortex and 15 months in the hippocampus [16]. Thus, the duration during which clopidogrel exerts its functional effects on the P2Y12 receptor might differ between microglia and platelets.

Notably, the P2Y12 receptor affects postoperative pain intensity and/or sensitivity of opioid analgesics through its antagonistic effect on microglia at the spinal cord and/or the PAG. Hence, the microglial P2Y12 receptor in the central nervous system would be a novel candidate for drug development targeting pain relief and/or preventing and treating opioid tolerance. However, this study was conducted as a retrospective chart review with a limited number of patients. To confirm our present findings, a prospective study with a larger number of participants needs to be performed.

## 4. Materials and Methods

This study was approved by the Ethical Review Board of the Faculty of Medicine, University of Tokyo (ID: 3678), and was registered at University Medical Information Network (12665). Opt-out consents were obtained from all participants.

### 4.1. Participants

We retrospectively reviewed the pharmacy and medical records of 27 patients with a history of cardiovascular diseases who were prescribed antiplatelet drugs and who underwent laparoscopic abdominal surgery during the study period (April 2014 through October 2016). All patients were administered general anaesthesia in combination with epidural anaesthesia. Postoperative pain was treated with a continuous epidural infusion of local anaesthetics (0.2% ropivacaine 120 mL, 4 mL/h) and intravenous fentanyl (10–30 µg/h). In the case of worsening pain, either bolus intravenous fentanyl (10–30 µg/dose) or NSAIDs (oral diclofenac, oral loxoprofen, or intravenous flurbiprofen) were administered. No protocols were available regarding the administration of opioid dosages or NSAIDs, and the attending physicians with expertise in postoperative pain management adjusted opioid or NSAIDs doses for individual patients at their discretion. Patients were divided into two groups based on clopidogrel use: one group was administered clopidogrel (CLP, *n* = 10), and the other group was given aspirin (ASP, *n* = 17); both groups had ceased antiplatelet drug usage 14 days prior to surgery. This time constraint was predetermined, considering the following factors: clopidogrel is a thienopyridine prodrug, whose active metabolite acts as an irreversible antagonist of the P2Y12 receptor. Following repeated clopidogrel administration, inhibition of platelet aggregation reaches a steady state. After clopidogrel administration is discontinued, platelet aggregation gradually returns and completely recovers to baseline values, generally within 14 days. Following a single systemic clopidogrel dose, its serum concentration reaches a peak in approximately 0.5 to 3 h in healthy subjects, disappearing mostly within 24 h. Reportedly, this time course of a single systemic clopidogrel administration in rats is similar to that in humans [17]. Additionally, a single systemic administration of clopidogrel has demonstrated anti-allodynic effects in a rat model of neuropathic pain by suppressing spinal microglia [18]. The anti-allodynic effect of clopidogrel on the spinal microglia starts at 2 h, lasting 6 h, and disappears by 8 h. Notably, this time course resembled that observed for the antiplatelet aggregation activity of a single systemic clopidogrel administration. Hence, we estimated the effect of clopidogrel on spinal microglia after reaching stable states by repeated systemic administration, and we established the time of clopidogrel cessation as 14 days before surgery. Based on the medical records, we confirmed that the postoperative pain intensities as measured on the 11-point NRS checked once daily from immediately after surgery until POD 3 scored less than 4. Furthermore, we confirmed the descriptions in daily records within 7 postoperative days, during which postoperative pain was well-managed. We collected data regarding NSAIDs use and the total dosages of opioids within 7 postoperative days (converted to intravenous fentanyl equivalents (mg)), required to manage postoperative pain under good control.

### 4.2. Statistical Analysis

Postoperative pain measured using NRS immediately after surgery and within 3 postoperative days was compared between CLP and ASP groups. The numbers of patients who used NSAIDs were counted and compared between the groups. The use of postoperative analgesics (opioids and NSAIDs) to manage postoperative pain during 7 postoperative days was compared between the two groups. These comparisons were performed using the Mann–Whitney test or chi-square test as suitable, and results with P-value < 0.05 were considered statistically significant.

## 5. Conclusions

The preliminarily findings of this retrospective chart review suggest that the P2Y12 receptor possibly mediates the signal transduction in postoperative nociceptive pain and enhances opioid-mediated analgesia.

## Figures and Tables

**Table 1:** Characteristics of patients in ASP and CLP. ASP, aspirin; CLP, clopidogrel

Groups	Male (%)	Age	BMI (Body Mass Index)	NSAIDs (%)
ASP group (N = 17)	94	74	22.9	60
CLP group (N = 10)	90	75	24.2	65
P-value	0.99	0.90	0.75	0.99

NSAIDs, non-steroidal anti-inflammatory drugs.

## References

[1] De Cosmo G., Aceto P., Gualtieri E., Congedo E. (2009). Analgesia in thoracic surgery: Review. Minerva Anesthesiol..

[2] Pisani M.A., Kong S.Y., Kasl S.V., Murphy T.E., Araujo K.L., Van Ness P.H. (2009). Days of delirium are associated with 1-year mortality in an older intensive care unit population. Am. J. Resp. Crit. Care Med..

[3] Bartels K., Mayes L.M., Dingmann C., Bullard K.J., Hopfer C.J., Binswanger I.A. (2016). Opioid use and storage patterns by patients after hospital discharge following surgery. PLoS ONE.

[4] Inoue K. (2006). The function of microglia through purinergic receptors: Neuropathic pain and cytokine release. Pharmacol. Ther..

[5] Inoue K. (2002). Microglial activation by purines and pyrimidines. Glia.

[6] Haynes S.E., Hollopeter G., Yang G., Kurpis D., Dailey M.E., Gan W.B., Julius D. (2006). The P2Y12 receptor regulates microglial activation by extracellular nucleotides. Nat. Neurosci..

[7] Sumitani M., Nishizawa D., Nagashima M., Ikeda K., Abe H., Kato R., Ueda H., Yamada Y. (2018). Japanese TR-Cancer Pain research group. Association Between Polymorphisms in the Purinergic P2Y12 Receptor Gene and Severity of Both Cancer Pain and Postoperative Pain. Pain Med..

[8] Miura G., Ariyoshi N., Sato Y., Yamaguchi H., Iwata Y., Fujimoto Y., Kobayashi Y., Ishii I. (2014). Genetic and non-genetic factors responsible for antiplatelet effects of clopidogrel in Japanese patients undergoing coronary stent implantation: An algorithm to predict on-clopidogrel platelet reactivity. Thromb. Res..

[9] Inoue K., Tsuda M. (2018). Microglia in neuropathic pain: Cellular and molecular mechanisms and therapeutic potential. Nat. Rev. Neurosci..

[10] Yu T., Zhang X., Shi H., Tian J., Sun L., Hu X., Cui W., Du D. (2019). P2Y12 regulates microglia activation and excitatory synaptic transmission in spinal lamina II neurons during neuropathic pain in rodents. Cell Death Dis..

[11] Lori N.E., Anne Z.M. (2019). Inflammatory Mediators of Opioid Tolerance: Implications for Dependency and Addiction. Peptides.

[12] Kolesnikov Y., Gabovits B., Levin A., Voiko E., Veske A. (2011). Combined catechol-O-methyltransferase and mu-opioid receptor gene polymorphisms affect morphine postoperative analgesia and central side effects. Anesth. Analg..

[13] Campa D., Gioia A., Tomei A., Poli P., Barale R. (2008). Association of ABCB1/MDR1 and OPRM1 gene polymorphisms with morphine pain relief. Clin. Pharmacol. Ther..

[14] Busch R., Neese R.A., Awada M., Hayes G.M., Hellerstein M.K. (2007). Measurement of cell proliferation by heavy water labeling. Nat. Protoc..

[15] Macallan D.C., Wallace D.L., Zhang Y., Ghattas H., Asquith B., de Lara C., Worth A., Panayiotakopoulos G., Griffin G.E., Tough D.F. (2005). B-cell kinetics in humans: Rapid turn over of peripheral blood memory cells. Blood.

[16] Tay T.L., Mai D., Dautzenberg J., Fernandez-Klett F., Lin G., Datta M., Drougard A., Stempfl T., Ardura-Fabregat A., Staszewski O. (2017). A new fate mapping system reveals context-dependent random or clonal expansion of microglia. Nat. Neurosci..

[17] Pravix^®^, Ethical Drug Package Insert by Japanese Pharmaceuticals and Medical Devices Agency. https://www.info.pmda.go.jp/go/pack/3399008F1025_1_26/?view=frame&style=XML&lang=ja.

[18] Tozaki-Saitoh H., Tsuda M., Miyata H., Ueda K., Kohsaka S., Inoue K. (2008). P2Y12 receptors in spinal microglia are required for neuropathic pain after peripheral nerve injury. J. Neurosci..

